# The effect of adjunctive intravitreal conbercept at the end of diabetic vitrectomy for the prevention of post-vitrectomy hemorrhage in patients with severe proliferative diabetic retinopathy: a prospective, randomized pilot study

**DOI:** 10.1186/s12886-020-1321-9

**Published:** 2020-02-03

**Authors:** Tingting Jiang, Junxiang Gu, Peijun Zhang, Wenwen Chen, Qing Chang

**Affiliations:** 1 Department of Ophthalmology, Eye and ENT Hospital, Fudan University, 83 Fenyang Road, Shanghai, 200031 China; 2 Shanghai Key Laboratory of Visual Impairment and Restoration, Shanghai, 200031 China; 3 Key Laboratory of Myopia of National Health Commission, Fudan University, Shanghai, China; 4 Key Laboratory of Myopia, Chinese Academy of Medical Sciences, Shanghai, China

**Keywords:** Diabetic retinopathy, Vitrectomy, Vitreous hemorrhage, Conbercept, Macular edema

## Abstract

**Background:**

To investigate the effect of intravitreal conbercept (IVC) injections on the incidence of postoperative vitreous hemorrhage (VH) in eyes undergoing surgery for severe proliferative diabetic retinopathy.

**Methods:**

This was a pilot prospective, comparative, and randomized study. Thirty patients, who underwent vitrectomy for severe proliferative diabetic retinopathy, were assigned randomly to either group 1 (intravitreal conbercept [IVC] injection at the end of pars plana vitrectomy) or group 2 (no IVC injection). Postoperative follow-up was performed on the first day, first week, first month, third month, sixth month and first year after surgery. The primary outcome was the incidence of postoperative VH. Secondary outcomes were the initial time of vitreous clearing (ITVC), best-corrected visual acuity (BCVA) and central retinal thickness (CRT) after surgery.

**Results:**

A total of 30 eyes, from 30 patients, were included. Fifteen eyes were enrolled in the IVC group and fifteen in the control group. The incidence of early and late postoperative VH was not significantly different between the control and IVC groups. ITVC was shorter in the IVC group than that in the control group, but this was not significant (7.38 ± 10.66 vs 13.23 ± 17.35, *P* = 0.31). Final BCVA, 1 year after surgery, showed significant improvement compared to baseline in both groups. However, analysis of the BCVA at any postoperative visit after surgery showed no significant differences between the two groups. There were two cases of recurrent VH identified at 3 and 6 months after surgery in each group, requiring a second round of surgery. Foveal thickness was significantly different between the two groups at the 3-month, 6-month and 1-year follow-up visits.

**Conclusions:**

In this pilot study, the effect of IVC injection in reducing the incidence of postoperative VH after diabetic vitrectomy at the end of vitrectomy was not shown.

**Trial registration:**

The study was registered with the Chinese Clinical Trial Registry. (Reference Number: ChiCTR1800015751).

## Background

Recurrence non-clearing vitreous hemorrhage (VH) is a major issue after undergo vitrectomy for proliferative diabetic retinopathy (PDR). The complication could delay visual recovery outcome and require additional surgery. Its reported PDR has been associated with a 29 to 75% incidence of VH recurrent ranges [1] during the initial postoperative period, and a 20 to 30% in longer follow-up periods. The systematically antifibrinolytic administration [2, 3], and the intravitreal infusion of short-acting gas, are the common treatment to reduce the incidence of VH [4, 5], but the clinical outcome are unsatisfactory. Recently, intravitreal anti-vascular endothelial growth factor (VEGF) drugs, as an adjunctive treatment, for complicated proliferative diabetic retinopathy (PDR) has been advocated [6, 7]. The pretreatment anti-VEGF agents for complicated PDR patients before vitrectomy could make surgery easier, reduce intraoperative bleeding and endodiathermy, shorter the surgery duration, and also reduce incidences of iatrogenic retinal breaks and incidence of early recurrent vitreous hemorrhage, also make quicker absorption of recurrent VH [8]. There are many reports in the literature investigating the effects of preoperative injection; however, there are very few studies evaluating the effects of intravitreal anti-VEGF agents at the end of surgery for postoperative VH in patients with PDR. However, the results of such procedures remain controversial. Some authors have reported that when injected intraoperatively, anti-VEGF drugs could result in a reduction in the incidence of early recurrent VH and quicker absorption of recurrent VH [9–11]. Other studies, have reported the opposite effects [12–14]. Conbercept (Chengdu Kanghong Biotech Co., Ltd., Sichuan, China) is a type of anti-VEGF agent, which has been widely used for curing patients with age-related macular degeneration (AMD) and macular edema and has been reported to be an effective adjunct when injected before vitrectomy for PDR [15]. The aim of the present study was to investigate the role of conbercept in the prevention of postoperative vitreous hemorrhage when injected at the end of vitrectomy for severe PDR.

## Methods

This was a pilot prospective, randomized clinical trial. The study followed the tenets of the Declaration of Helsinki and was approved by the Research Ethics Committee of Eye and ENT Hospital, Fudan university. Informed consent was obtained from all patients prior to surgery. The study was registered with the Chinese Clinical Trial Registry. (Reference Number: ChiCTR1800015751).

We conducted a pilot prospective, randomized clinical trial on a series of patients who required vitrectomy for severe PDR. Randomization was carried out according to randomized table. The indications for surgery were PDR-related complications, such as non-clearing vitreous hemorrhage, fibrovascular proliferation, and macula-involving, or macula-threatening, tractional retinal detachment. Patients were excluded if there was a previous history of vitreoretinal surgery, intravitreal injection of long-acting gas or silicone oil at the end of surgery, a history of ocular diseases other than diabetic VH, a history of intravitreal anti-VEGF within the 3 previous months, ocular surgery within the previous 6 months, uncontrolled hypertension, a history of coagulopathy, and a follow-up period less than 12 months.

In preoperative examinations, all patients underwent best-corrected visual acuity (BCVA) assessment, slit-lamp biomicroscopy, intraocular pressure (IOP) measurement using applanation tonometry, fundus examination by indirect ophthalmoscopy, fundus photography and B-scan ultrasonography. Postoperative visits were performed on the first day, and then in the first week, first month, third month, sixth month and then after 1 year. At each postoperative visit, in addition to the examinations mentioned above, optical coherence tomography (OCT) was performed. Fluorescein angiography was performed 6 months after surgery. Vitreous hemorrhage was graded by indirect ophthalmoscopic fundus examination according to a grading system (Table 1) [9]. Early postoperative vitreous hemorrhage (POVH) was defined as VH occurring between 1 week and 1 month after surgery while late POVH was defined as VH occurring between 4 weeks and 12 months after surgery. Recurrent VH was defined as a new episode of VH (grade 1 or higher) occurring more than 1 week after surgery. Assessors were masked to group.

**Table 1:** Grading system for vitreous hemorrhage

Description	Grade
No vitreous hemorrhage	0
Mild vitreous hemorrhage with visible fundus details, but difficult to evaluate the retinal nerve fiber layer or small vessels	1
Moderate vitreous hemorrhage with visible optic disc and large vessels	2
Severe vitreous hemorrhage with faint fundus reflex, only optic disc visible	3
Very severe vitreous hemorrhage with no fundus reflex and no view of the fundus	4

The surgical procedures were performed by a single surgeon. All patients underwent 25-gauge transconjunctival vitrectomy. Total vitrectomy was performed in every case; peripheral vitrectomy was completed with scleral indentation under a wide-angle visualization system. Triamcinolone acetonide was used to ensure that the posterior hyaloid was eliminated. Laser photocoagulation was administered to any areas of untreated retina to complete pan-retinal photocoagulation. At the conclusion of each case, retinal breaks and residual sources of bleeding were assessed. Sutures were placed in leaking sclerotomy sites in order to avoid postoperative hypotony. Patients in the control group did not receive intravitreal conbercept (IVC), whereas those in the intervention group received IVC at the end of surgery (0.5 mg in 0.05 ml). Whether injection or not depended on the randomized results.

Preoperative data, including baseline demographics (age and gender), systemic factors (hypertension and hemoglobin A1c at the time of surgery) and ophthalmic factors (previous history of pan-retinal photocoagulation, lens status) were collected for each patient. During surgery, the extent of vitreoretinal adhesion was assessed according to a grading system (Table 2) [9] and samples of vitreous were taken for angiogenic factor testing.

**Table 2:** Grading for the extent of vitreoretinal adhesion

Adhesion	Grade
Absence of any adhesion	0
adhesion at 3 sites of fewer	1
broad adhesion at 1 site or more or adhesion at the disc, macula, and vascular arcade	2
vitreoretinal adhesion extending to the periphery	3

The primary outcome measure was the incidence of postoperative VH. Secondary outcome measures were the initial time of vitreous clearing (ITVC), BCVA and central retinal thickness after surgery. The ITVC was defined as the interval (the number of days) for VH (grade 1 or higher) observed at postoperative day 1 to clear up completely.

When the distribution of data was normal, differences between the two groups were assessed for statistical significance using the Student’s t test. Nonparametric analysis was selected when the distribution of data differed significantly from normal. Differences between groups were determined using chi-square analysis or Fishers’ Exact test for categorical data. Statistical analyses were performed using SPSS statistical software (version 20.0; SPSS Inc., Chicago, IL). For all statistical tests, *P* < 0.05 was considered significant.

## Results

**Fig. 1:**
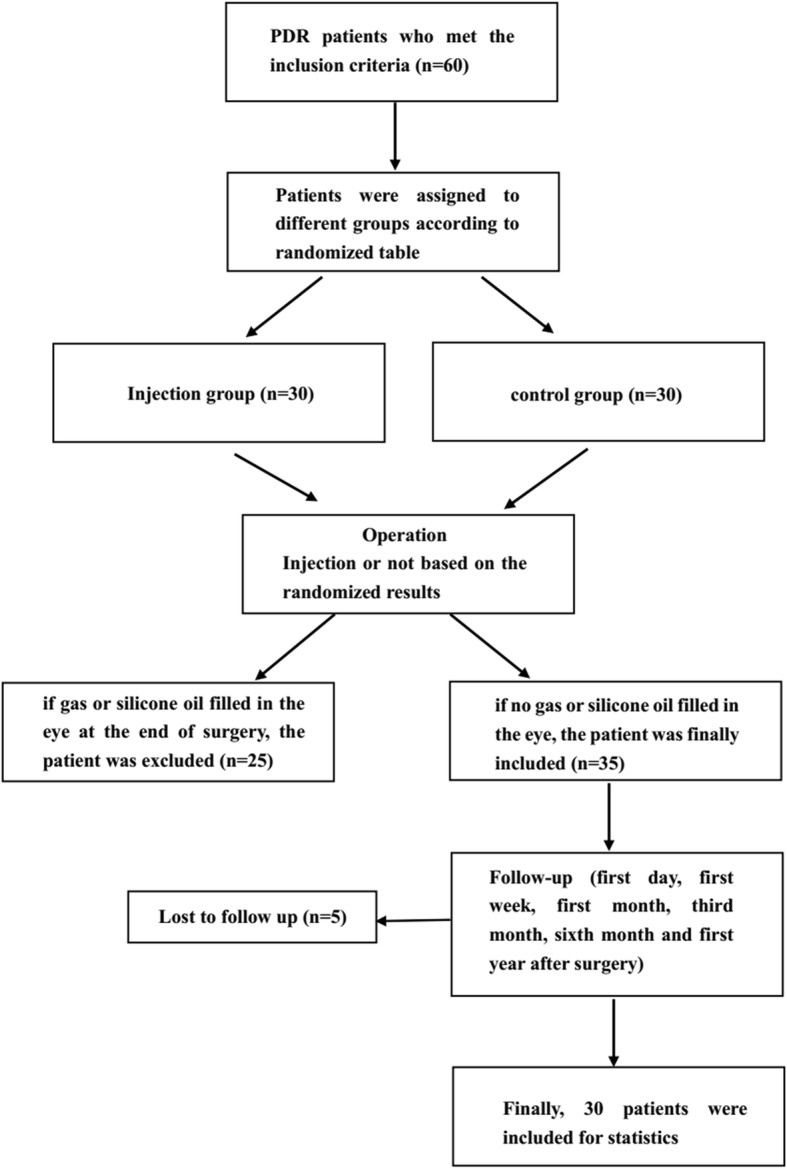
The flow diagram of enrollment

Finally, a total of 30 eyes, from 30 patients who met the inclusion criteria, were included in this study (15 eyes in group 1, 15 eyes in group 2). The flow diagram of enrollment was shown in Fig. 1, which contained the details. As detailed in Table 3, there were no statistically significant differences in terms of age, gender, hypertension, hemoglobin A1c, years of diabetes, previous history of panretinal photocoagulation, lens status, baseline BCVA, or the extent of vitreoretinal adhesion, when compared between the two groups. Due to masking of the retina by vitreous hemorrhage, we evaluated the range of the vasoproliferative membranes during surgery. In the IVC group, the extent of the vasoproliferative membranes was 1.33 ± 1.4 quadrants compared with 1.2 ± 1.47 quadrants in the control group (*P* = 0.8) (Table 4).

**Table 3:** Baseline patient demographic data

	Intraoperative intravitreal conbercept (*n* = 15) (mean ± SD)	Control group (*n* = 15) (mean ± SD)	*P* value
Mean age, (yrs)	55.54 ± 9.94	53.5 ± 9.59	0.57
Gender (male/female)	6/9	10/5	0.3
Duration of diabetes, (y)	13.19 ± 8.08	9.88 ± 8.52	0.32
Hypertension	7/15(46.7%)	5/15(33.3%)	0.71
Lens status (Phakic/pseudophakic)	12/3	14/1	0.6
IOP (mmHg)	12.46 ± 3.26	12.3 ± 1.44	0.88
HbA1c at time of surgery	6.65 ± 1.13	6.8 ± 1.00	0.7
Preoperative pan-retinal photocoagulation history			0.9
None	5(33.3%)	4(26.7%)	
Incomplete	6(40%)	6(40%)	
complete	4(26.7%)	5(33.3%)	
Preoperative VH grades			0.47
1	1(6.7%)	2(13.3%)	
2	5(33.3%)	2(13.3%)	
3	3(20%)	6(40%)	
4	6(40%)	5(33.3%)	
Preoperative BCVA (logMAR)	2.02 ± 0.8	1.62 ± 0.69	0.18
Extent of vitreoretinal adhesion grade			0.91
0	2(13.3%)	2(13.3%)	
1	3(20%)	4(26.7%)	
2	10(66.7%)	9(60%)	
3	0	0	

*VH* Vitreous hemorrhage

*BCVA* Best corrected visual acuity

**Table 4:** The range of the neovascularization membrane

	IVC group	Control group	*P*
The neovascularization membrane(quadrants)	1.33 ± 1.4	1.2 ± 1.47	0.8

The incidence of early and late postoperative VH was not significantly different when compared between the control and the IVC groups (Table 5). The ITVC was shorter in the IVC group than in the control group, but this was not statistically significant (7.38 ± 10.66 vs 13.23 ± 17.35, *P* = 0.31). Analysis of BCVA at 1 day, 1 week, 1 month, 3 months, 6 months and 1 year after surgery showed no significant difference between the two groups. In the IVC group, as illustrated in Table 6, BCVA (logMAR) increased significantly from 2.02 ± 0.8 at baseline to 0.67 ± 0.64 at 6 months(*P* = 0.001) and to 0.56 ± 0.52 at 1 year(P<0.001). In the control group, BCVA (logMAR) increased significantly from 1.62 ± 0.69 at baseline to 0.74 ± 0.53 at 6 months(*P* = 0.003) and 0.42 ± 0.37 at 1 year(P<0.001). Final BCVA, 1 year after surgery, showed significant improvement compared with baseline in both of the groups. There were two cases of recurrent vitreous hemorrhage occurring in the 3 and 6 months after surgery in each group; these patients all underwent a second round of surgery. Foveal thickness (μm) showed a statistically significant difference between the two groups at the 3-month, 6-month and 1-year follow-up (Table 7; 280.83 ± 66.45 vs 403.08 ± 161.77 at 3 months (*p* = 0.029), 258.64 ± 48.45 vs 388.42 ± 128.78 at 6 months (*p* = 0.005), 257.83 ± 45.47 vs 345 ± 131.75 at 12 months (*p* = 0.04). Final foveal thickness, 1 year after surgery, showed significant improvement compared to baseline in the IVC group. The concentrations of angiogenic factors in the vitreous are shown in Table 8. Basic fibroblast growth factor (bFGF) and leptin levels from the vitreous of patients in the IVC group were significantly higher (*p* < 0.05) than those in the control group.

**Table 5:** The occurrence of postoperative hemorrhage

Postoperative VH grade	IVC group (n)	Control group(n)	P value
Day 1			0.26
0	7	4	
1	0	2	
2	2	3	
3	1	4	
4	5	2	
First week			0.62
0	13	12	
1	0	0	
2	0	0	
3	2	3	
4	0	0	
First month			0.31
0	15	14	
1		1	
2			
3			
4			
Third month			0.37
0	14	14	
1			
2			
3		1	
4	1		
Sixth month			
0	14	14	
1			
2			
3	1	1	
4			
1 year			
0	15	15	
1			
2			
3			
4			

*IVC* Intraoperative intravitreal conbercept group

*VH* Vitreous hemorrhage

**Table 6:** Changes in BCVA from baseline to 1 year after surgery

BCVA (logMAR)	IVC group (mean ± SD)	Control group (mean ± SD)	*P* value
Preoperative	2.02 ± 0.8	1.62 ± 0.69	0.18
day1	2.04 ± 1.02	1.89 ± 1.02	0.70
1 week	1.09 ± 0.66	1.06 ± 0.47	0.89
1 month	0.75 ± 0.6	0.63 ± 0.36	0.55
3 months	0.79 ± 0.82	0.74 ± 0.54	0.86
6 months	0.67 ± 0.64	0.74 ± 0.53	0.78
1 year	0.56 ± 0.52	0.42 ± 0.37	0.43

*IVC* Intraoperative intravitreal conbercept group

*BCVA* Best corrected visual acuity

**Table 7:** Changes in CRT from baseline to 1 year after surgery

CRT (μm)	IVC group(mean ± SD)	Control group(mean ± SD)	*P* value
1 week	372.42 ± 143.99	379.64 ± 126.29	0.900
1 month	294.62 ± 66.03	390.08 ± 145.62	0.050
3 months	280.83 ± 66.45	403.08 ± 161.77	0.029
6 months	258.64 ± 48.45	388.42 ± 128.78	0.005
1 year	257.83 ± 45.47	345 ± 131.75	0.040

*IVC* Intraoperative intravitreal conbercept group

*CRT* Central retinal thickness

**Table 8:** The concentrations of cytokines in the vitreous

Cytokines	IVC group (pg/ml)	Control(pg/ml)	*P*
ANG2	307.71	196.46	0.37
Angiogenin	2479.21	2529.41	0.55
VEGF	304.7	270.78	0.65
bFGF	59.93	47.78	0.01
EGF	0.71	0.73	0.21
HGF	4308.85	3320.97	0.31
Leptin	26.54	10.96	<0.01
PDGF	7.32	6.29	0.85
PLGF	69.06	78.95	0.21

*IVC* Intraoperative intravitreal conbercept group

*ANG2* Angiopoietin-2

*VEGF* Vascular endothelial growth factor

*bFGF* Basic fibroblast growth factor

*EGF* Epidermal Growth Factor

*HGF* Hepatocyte growth factor

*PDGF* Platelet derived growth factor

*PLGF* Placenta growth factor

## Discussion

Literature shows that VH developed after pars plana vitrectomy in 29–75% of the patients reported [1, 16, 17]. Reactivation of fibrovascular tissue remnants in retinal sites may be the cause of early POVH, while sclerotomy site neovascularization or anterior hyaloidal fibrovascular proliferation may be the cause of late POVH [18]. It is well confirmed, as VEGF is one of the crucial major angiogenic factors, it involved in the proliferative diabetic retinopathy. Studies have reported a rising angiogenic factors concentration, such as VEGF, in patients with diabetic retinopathy after ocular surgery because of the surgical trauma with its induced inflammation [19–23].

In spite of the pretreatment of anti-VEGF agents before vitrectomy to the complicated cases of PDR could easier surgery, we believe that blockage of VEGF surge by administering the drug at the end of surgery, is better than before the surgery. It could provide more effective path for reducing complications such as POVH because it helps to avoid vitrectomy induced pretreatment drugs wash away [24, 25]. Relatively fewer studies have investigated the use of intravitreal anti-VEGF agents at the end of surgery; unfortunately, such studies yielded controversial findings. Some of these studies [9–11] reported that the use of anti-VEGF agents could significantly reduce the incidence of postoperative VH. Others, however, suggested that intravitreal anti-VEGF had no beneficial effect upon the incidence of postoperative VH when injected at the end of surgery [12–14]. While most of these studies were conducted using bevacizumab, our present study used conbercept, a recombinant and soluble protein composed of the VEGF receptor and the Fc portion of immunoglobulin G. Conbercept functions by competitively inhibiting the binding of VEGF with its receptor by blocking multiple targets: VEGF-A, VEGF-B and placental growth factor (PLGF) [26]. Therefore, this drug is relatively stable and long lasting, in comparison with that of monoclonal antibodies. Furthermore, preclinical studies have documented a higher affinity of conbercept for VEGF than bevacizumab [27]. However, the effect of this drug on the rate of postoperative VH in patients with severe PDR when administered at the end of the vitrectomy has not been evaluated. This pilot prospective randomized study was conducted to investigate the efficacy of an intraoperative intravitreal injection of conbercept in cases of diabetic vitrectomy.

In our study, we found no significant differences in the incidence of either early or late postoperative VH when compared between the two groups. The incidences of early postoperative VH were 13.3% in the IVC group and 20% in the control group; according to previous reports, these rates were 5–38.2% in an IVB (intravitreal injection of bevacizumab) group and 13–36.8% in a control group [9, 10, 13, 14]. In our study, the rates of early POVH appeared to be lower than some previous reports. This difference might de due to differences related to anti-VEGF strength when compared between conbercept and bevacizumab, performing total vitrectomy with posterior hyaloid detachment and meticulous vitreous base cleaning. However, we should also take into account other reasons, such as differing baselines for systemic and ocular profile, and differences in sample size. The ITVC was 7.38 ± 10.66 in our IVC group, which was shorter than that in the control group (13.23 ± 17.35), although this was not statistically significant. In our study, two patients in each group experienced recurrent vitreous hemorrhage; this happened at 3-month and 6-month follow up. For both groups, the rate of late recurrent hemorrhage was 6.7%; all of these patients underwent a second round of surgery. The etiology of late recurrent postoperative VH is considered to involve fibrovascular ingrowth at the sclerotomy sites, along with anterior hyaloidal fibrovascular proliferation [28, 29]. However, in all the patients undergoing reoperation in our study, the surgeon checked the entry site carefully, and found no neovascularization at the sclerostomy sites through scleral indentation. Some patients just had simple vitreous cavity washout while others added retinal laser photocoagulation. We were careful to remove peripheral vitreous as much as possible, especially around the sclerostomy areas through deep scleral indentation during the first surgery. After the second surgery, no one has vitreous cavity hemorrhage again during the follow up. Therefore, not all late postvitrectomy hemorrhages for PDR are caused by entry site neovascularization. A high level of VEGF in the vitreous fluid has been identified as a significant risk factor for the outcomes of vitreous surgery in patients with PDR [30]. It is also well known that after vitrectomy, there is a VEGF surge acting as a stimulant for iris neovascularization or fibrovascular proliferation that may lead to VH [21]. Therefore, intravitreal anti-VEGF injection at the end of surgery, could inhibit VEGF elevation effectively, inhibiting retinal neovascularization, a potential cause for postoperative VH. Also, Inhibition of VEGF activity could stabilize the vascular permeability. However, faster vitreous clearance rates for medications in vitrectomized eyes have been observed, the anti-VEGF drugs injection may only have their effects during the early postoperative period. In addition, VH is closely related to blood glucose control [21]; thus a single dose may not be sufficient to prevent the onset of late VH.

We found no beneficial effect of IVC on postoperative VH and ITVC. However, caution should be taken in comparing our results directly to those of previous studies because detailed baseline information, such as indications for surgery, the severity of fibrovascular proliferation, and systemic factors were different. This may be due to the inclusion of severe proliferative diabetic retinopathy patients in our study. In addition to VH, most of the patients in our study had severe fibrovascular proliferation and many had firm adhesions between the vitreous and retina. Due to masking of the retina by VH, we evaluated the range of the vasoproliferative membranes during surgery. In the IVC group, the extent of the vasoproliferative membranes was 1.33 ± 1.4 quadrants compared with 1.2 ± 1.47 quadrants in the control group (*P* = 0.8). Such information was not provided in previous studies.

Pathological development of PDR is a complex process, which involves several growth factors, such as VEGF, bFGF (basic fibroblast growth factor), hepatocyte growth factor (HGF), interleukin-1β (IL-1β), tumor necrosis factor-α (TNF-α), interleukin-8 (IL-8),, monocyte chemoattractant protein-1 (MCP-1), and platelet-derived growth factor (PDGF) [31–33]. In this study, we measured the concentrations of a range of cytokines in vitreous samples taken from our patients. The levels of bFGF and leptin from the vitreous of patients in the IVC group were significantly higher (*p* < 0.05) than those in the control group. Although there was no statistical significance, the levels of VEGF and angiopoietin (ANG) 2 in the vitreous were higher in patients from the IVC group than those from the control group. As reported that leptin stimulated ischemia-induced retinal neovascularization, possibly by endothelial VEGF upregulation [34]. bFGF is produced and stored in epiretinal membranes, it may also play a part in the control of proliferative at the vitreoretinal interface [35]. Moreover, as a risk factor, intraocular VEGF levels before surgery have been used for predicting the outcome, or complications of PDR surgery, such as early postoperative VH [36, 37]. Same time, several cytokines and growth factors are also thought to as a correlated risk factor for the severity of PDR [38]. To some extent, our results indicated that patients in the injection group were relatively more serious than those in the control group. Therefore, our results should be interpreted by taking this bias into account.

In our study, postoperative VA significantly increased in both groups but did not differ significantly between the groups at any of the postoperative visits. This is consistent with previous reports [9–12, 14]. One study reported that intraoperative treatment with intravitreal bevacizumab showed the best visual recovery at the end of 6 months compared with other groups [13]. However, this report was a retrospective and non-randomized study, and may thus have inherently caused bias in the results.

Macular edema (ME) sometimes persists or recurs in many vitrectomized eyes with PDR. In many previous studies, the presence of macular edema, another significant factor affecting visual acuity in diabetic retinopathy, was not analyzed. In this study, we measured central retinal thickness (CRT) at each postoperative visit. Foveal thickness (μm) measurements decreased significantly in the IVC group. Furthermore, there were significant differences in CRT between the two groups at the 3-month, 6-month and 12-month follow-up visits. These results were confusing because it is well known that anti-VEGF drugs such as conbercept have a relatively short half-life, especially in vitrectomized eyes. However, there was not an upwards trend in mean macular thickness; even when measured 6 months after surgery. This is not consistent with the results of a previous study which suggested that conbercept had limited effect on reducing macular edema after surgery [39]. This may be due to the small sample size of our study. Meanwhile, in our study, there is a discrepancy between the foveal thickness and the vision acuity in the injection group. The reduction in macular thickness was not accompanied by significant improvement in vision acuity. The decreasing CRT was associated with macular atrophy and destruction of the outer structure of the retina, which all lead to poor vision prognosis.

The present study reports the outcomes of the intraoperative intravitreal injection of conbercept after diabetic vitrectomy, a topic which has not been extensively investigated previously. The limitation of this study is the small number of patients involved. However, this study features a randomized prospective design, long term follow-up and surgery was carried out by a single surgeon. When we started the trial, we planned to enroll more people than this (details in Fig. 1). But proliferative diabetic retinopathy of the patients in our study were very severe, as a result, many of them undergone intravitreal injection of long-acting gas or silicone oil at the end of surgery and these patients had to be excluded. Meanwhile, due to lost to follow up, finally, only 30 people were included for statistics. In our opinion, although the sample size is small, the obtained data could provide a preliminary result in this pilot study. At present, this randomized and prospective study is still going on, and a more powered conclusion with large sample sizes will be reported in the future.

## Conclusions

In conclusion, in this pilot study, intraoperative IVC did not appear to have an encouraging effect on the rate of postoperative VH and visual recovery. Future randomized and prospective studies, with large sample sizes, are now necessary to further investigate the effect of conbercept at the end of vitrectomy for diabetic retinopathy. The results of this current study should provide a useful reference for future trials.

## Data Availability

The datasets used and/or analysed during the current study available from the corresponding author on reasonable request.

## Abbreviations

AMD: Age-related macular degeneration
ANG: Angiopoietin
BCVA: Best-corrected visual acuity
bFGF: Basic fibroblast growth factor
CRT: Central retinal thickness
HGF: Hepatocyte growth factor
IL-1β: Interleukin-1β
IL-8: Interleukin-8
IOP: Intraocular pressure
ITVC: Initial time of vitreous clearing
IVB: Intravitreal injection of bevacizumab
IVC: Intravitreal conbercept
MCP-1: Monocyte chemoattractant protein-1
ME: Macular edema
OCT: Optical coherence tomography
PDGF: Platelet-derived growth factor
PDR: Proliferative diabetic retinopathy
PLGF: Placental growth factor
POVH: Postoperative vitreous hemorrhage
TNF-α: Tumor necrosis factor-α
VEGF: Vascular endothelial growth factor
VH: Vitreous hemorrhage
